# Telomere Length Increase in HIV/HCV-Coinfected Patients with Cirrhosis after HCV Eradication with Direct-Acting Antivirals ^†^

**DOI:** 10.3390/jcm9082407

**Published:** 2020-07-28

**Authors:** Silvia Molina-Carrión, Óscar Brochado-Kith, Juan González-García, Juan Berenguer, Cristina Díez, Elba Llop, Victor Hontañón, Luis Ibañez-Samaniego, Maria Luisa Montes, Salvador Resino, Amanda Fernández-Rodríguez, María Ángeles Jiménez-Sousa

**Affiliations:** 1 Instituto de Salud Carlos III, Centro Nacional de Microbiología, Unidad de Infección Viral e Inmunidad, Carretera Majadahonda-Pozuelo, Km 2.2, Majadahonda, 28220 Madrid, Spain; Silsu22@outlook.com (S.M.-C.); obrochado@isciii.es (Ó.B.-K.); jimenezsousa@isciii.es (M.Á.J.-S.); 2 HIV Unit, Internal Medicine Service, La Paz University Hospital, 28046 Madrid, Spain; juangonzalezgar@gmail.com (J.G.-G.); victor.hontanon@gmail.com (V.H.); mmontesr2001@yahoo.es (M.L.M.); 3 Infectious Disease/HIV Unit, Gregorio Marañón G. University Hospital, 28007 Madrid, Spain; jbb4@me.com (J.B.); crispu82@gmail.com (C.D.); 4 Servicio de Gastroenterología, Hospital Universitario Puerta de Hierro, Majadahonda, 28222 Madrid, Spain; elballop@gmail.com; 5 Servicio de Digestive, Hospital General Universitario “Gregorio Marañón”, 28007 Madrid, Spain; lisamaniego@gmail.com; 6 Department of Medicine, Alfonso X el Sabio, Villanueva de la Cañada, 28691 Madrid, Spain

**Keywords:** coinfection, hepatitis C, HIV, DAAs, telomeres, cirrhosis, decompensation

## Abstract

Introduction: Human immunodeficiency virus (HIV) infection and cirrhosis are associated with a senescent phenotype that decreases telomere length. We evaluated the impact of hepatitis C virus (HCV) elimination on telomere length in patients with advanced HCV-related cirrhosis after sustained virological response (SVR), with all-oral direct-acting antiviral agents (DAAs). Methods: Prospective study of 60 HIV/HCV-coinfected and 30 HCV-monoinfected patients with advanced HCV cirrhosis (liver decompensation or liver stiffness measurement (LSM) ≥ 25 kPa, hepatic liver pressure gradient (HVPG) ≥ 10 mmHg, or Child–Pugh–Turcotte (CPT) ≥ 7). The relative telomere length (RTL) was quantified by real-time multiplex PCR (MMqPCR) on peripheral blood mononuclear cells at baseline and 48 weeks after HCV treatment. Generalized linear models (GLMs) adjusted for the most relevant clinical and epidemiological variables and mixed GLMs were used. Results: In comparison with HCV-monoinfected patients, HIV/HCV-coinfected patients were younger (*p* < 0.001), had lower body mass index (BMI) (*p* = 0.002), and had been exposed less frequently to interferons (*p* = 0.011). In addition, they were more frequently men (*p* = 0.011), smokers (*p* = 0.005), prior intravenous drug users (IVDUs) (*p* < 0.001), and alcohol abusers (*p* = 0.005). RTL was significantly lower in HIV/HCV-coinfected patients than in HCV-monoinfected patients, both at baseline (*p* < 0.001), and at the end of follow-up (*p* = 0.032). A significant RTL increase over time was found only for HIV/HCV-coinfected patients (*p* < 0.001), especially in those patients with compensated cirrhosis (*p* < 0.001). Conclusion: HCV eradication with all-oral DAAs was associated with an increase in telomere length in HIV/HCV-coinfected patients with advanced cirrhosis, particularly in compensated patients. This finding suggests that HCV clearance may have implications in age-related conditions in this population group.

## 1. Introduction

Telomeres are repetitive nucleotide sequences at the end of chromosomes that protect against genome instability. The absence or inhibition of reverse telomerase transcriptase activity leads to a loss of a portion of the telomere with each cell division, until the length of the telomeres reaches a critical size, inducing cell senescence and apoptosis. However, some dividing cells, such as lymphocytes, express telomerase, which can slow or even reverse telomere shortening. Telomere length reflects the replicative potential of cells, which decreases with age. It also is a predictor of mortality and comorbidities in the general population and in people living with human immunodeficiency virus (HIV) [1]. HIV promotes chronic immune activation, oxidative stress, inflammation, and an accelerating loss of telomere length in immune cells during the acute and chronic phase of infection [2]. Telomere reduction seems to occur from the first point of HIV infection, and may be maintained during HIV infection without antiretroviral therapy (ART) [3,4]. There is evidence that ART and control of HIV viremia have beneficial effects on telomere length [1]. However, most ART regimens contain nucleoside reverse transcriptase inhibitors (NRTIs), which could inhibit telomerase activity [5].

Chronic hepatitis C (CHC) leads to a decrease in telomere length of T-cells, which seems to be more related to the severity of liver disease than to the HCV infection itself [4,6,7]. In the liver, hepatocyte telomeres are shorter in cirrhotic patients [8]. HCV promotes a high renewal rate of infected hepatocytes, oxidative stress, and inflammation, which induces cellular senescence and fibrosis [9]. In blood, CHC is linked to T-cell exhaustion, characterized by telomere shortening [2]. Repetitive antigenic stimulation encourages immune cells to divide, and the resulting decrease in telomere length leads to CHC progression and the appearance of cirrhosis-associated immune dysfunction, characterized by elevated immune activation, inflammation, and immunosuppression [10].

Pegylated interferon plus ribavirin (PR) has been the gold standard of hepatitis C virus (HCV) therapy for many years, up until the introduction of direct-acting antivirals (DAAs). The new DAAs have revolutionized HCV therapy, with excellent antiviral efficacy and very high cure rates, being safe and effective in both HCV and HIV/HCV-coinfected patients [11]. The American Association form the study of liver diseases and the Infectious Diseases Society of America (AASLD–IDSA) HCV guidance recommends using the same general approach for treating HCV in patients with HCV monoinfection and HIV/HCV coinfection, but notes the importance of considering potential drug interactions with HIV antiretroviral medications [12]. Regarding the impact of DAA therapy on telomere size, telomere elongation has recently been described in HCV-monoinfected patients with cirrhosis who reached sustained virological response (SVR) after DAA treatment [11], but there is no evidence of telomere elongation in HIV/HCV-coinfected patients after HCV eradication with DAAs.

Here, we aimed to evaluate the impact of HCV elimination with all-oral DAAs on telomere length in HIV/HCV-coinfected patients with advanced, HCV-related cirrhosis.

## 2. Patients and Methods

### 2.1. Patients

We carried out a multicenter, prospective observational study on 90 patients with advanced HCV-related cirrhosis from the ESCORIAL cohort (see Appendix A) who started anti-HCV therapy with all-oral DAAs from January to December 2015. Samples were collected between January 2015 and June 2016. The study was conducted in accordance with the Declaration of Helsinki; all patients gave their written consent before enrollment, and the Research Ethics Committee of the Instituto de Salud Carlos III approved the study (CEI PI 41_2014).

The inclusion criteria were (1) plasma HCV RNA detectable by polymerase chain reaction (PCR); (2) advanced cirrhosis, defined by (i) prior history of liver decompensation (ascites, bleeding esophageal varices, hepatic encephalopathy), (ii) a Child–Pugh–Turcotte (CPT) score ≥7, (iii) liver stiffness ≥ 25 kPa, or (iv) a hepatic liver pressure gradient (HVPG) ≥10 mmHg; and (3) starting HCV treatment with all-oral DAAs. SVR was defined as an undetectable HCV RNA load 12 weeks after finalization of anti-HCV therapy. HIV/HCV-coinfected patients were on stable combination antiretroviral therapy (cART) for ≥6 months and had undetectable plasma HIV viral loads (<50 copies/mL). Hepatic decompensation was defined by prior history of liver decompensation (ascites, bleeding esophageal varices, or hepatic encephalopathy) or Child–Turcotte–Pugh (CTP) ≥ 7 at baseline.

**Figure 1:**
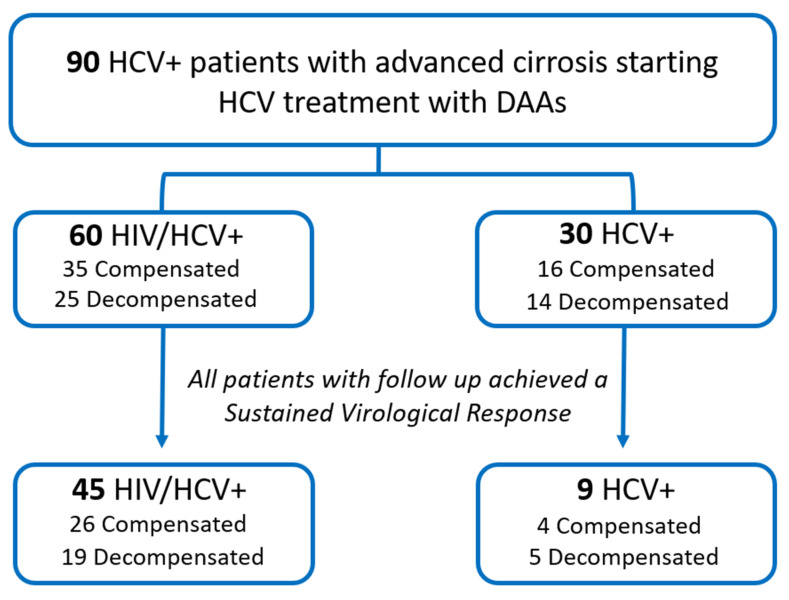
Flow chart of patient selection. Stratification according to decompensation is referred to at the baseline.

Sixty HIV/HCV-coinfected and 30 HCV-monoinfected patients were included at baseline, of which 45 HIV/HCV-coinfected and nine HCV-monoinfected patients completed the follow-up of the study at 48 weeks after DAA treatment completion, and achieved an SVR. Of the 45 HIV/HCV-coinfected patients with follow-up, 26 were compensated and 19 were decompensated at baseline (Figure 1).

In Spain, anti-HCV therapy is provided by hospital pharmacies and is covered by the National Health System. The decision to administer anti-HCV therapy and selection of the adequate regimen was taken by hepatologists or medical specialists in infectious disease at each institution.

### 2.2. Relative Quantification of Telomeres

Peripheral venous blood samples were collected in ethylenediaminetetraacetic acid (EDTA) tubes, and peripheral blood mononuclear cells (PBMCs) were isolated with Ficoll–Paque (GE Healthcare). DNA was extracted with the DNA Purification System Kit (Promega Wizard).

We performed a monochromatic multiplex real-time quantitative PCR (MMqPCR) assay for relative telomere length (RTL), based on the work of Cawthon et al. [13] and modified for a LightCycler 480 instrument (Roche) by Hsieh et al. [14].

Briefly, each MMqPCR reaction was performed with 7.5 µL of GoTaq qPCR Master Mix (Promega) with a final concentration of 1×, 0.15 µM of each of the four primers, 1 mM of dithiothreitol (DTT), and 20 ng of DNA. The thermal cycling profile was initiated with 95 °C enzyme activation (hot-start) incubation for 15 min. Next were two cycles of 94 °C for 15 s (2.2_C/s) and 49 °C for 15 s (2.2_C/s), and then 35 cycles of 94 °C for 15 s, 62 °C for 10 s, (2.2_C/s), 74 °C for 15 s, 84 °C for 10 s, and 88 °C for 15 s, with signal acquisitions at the end of the 74 °C and 88 °C stages. After cycling, a melting curve program was run, starting with a 95 °C incubation for 1 min, followed by continuous acquisitions every 0.2 °C from 45 °C to 95 °C (ramping at 0.11_C/s). All temperature ramping rates were set at 4.4_C/s or 2.2_C/s where indicated, except the melting curve, which was ramping at 0.11_C/s. The primer sequences were as follows: Tel_F = 5′-ACACTAAGGTTTGGGTTTGGGTTTGGGTTTGGGTTAGTGT-3′; Tel_R = 5′-TGTTAGGTATCCCTATCCCTATCCCTATCCCTATCCCTAACA-3′; HBB_F = 5′-CGGCGGCGGGCGGCGCGGGCTGGGCGGcttcatccacgttcaccttg-3′; and HBB_R = 5′-GCCCGGCCCGCCGCGCCCGTCCCGCCGgaggagaagtctgccgtt-3′. A standard curve was prepared from the DNA of a reference sample (1301, lymphoblast cell line), with concentrations ranging from 0.74 ng to 82 ng, and it was run in duplicate for each run, together with a negative control.

Fluorescence raw data was extracted for each amplicon, as previously described [14]. Fluorescence was captured at the different dissociation temperatures of the two amplicons. However, as the LightCycler instrument software does not allow dual-signal acquisition processing, several tools were used in order to convert and process separate acquisition data from telomeric DNA (T) and single copy genes (S) beta-globin, (HBB), as previously described by Hsieh et al. [14]. First, data were exported from the LightCycler instrument software in text format and imported into Microsoft Excel to split the 74 °C acquisition data from the 88 °C acquisition data. Subsequently, acquisition-delineated data were converted into grid format with the LC480Conversion Program (LC480cp; http://www.hartfaalcentrum.nl/index.php?main=files&fileName=LC480Conversion.zip&description=LC480Conversion:%20conversion%20of%20raw%20data%20from%20LC480&sub=LC480Conversion). Later, LinRegPCR [15] was used to perform baseline corrections and Ct calculations.

Subsequently, RTL was expressed as T normalized to the number of copies of S, obtaining a T/S ratio [14]. On each plate, the standard curve was calculated for each product by averaging the raw Ct values previously extracted. Ct values were plotted against the logarithm of the DNA concentration on an X/Y scatter plot, and the linear trend line was generated together with the equation in the form of *y* = *ax* + *b*, where *y* was the log (DNA) concentration value, *a* the slope, *x* the Ct value of each well, and *b* the intercept. The linear DNA data was obtained with the equation: T or S = 10 ^ (log (DNA)), which allows us to obtain the T and S values for the telomere product and the single copy gene, respectively. Each plate was normalized by the PCR efficiency of its standard curve. RTL was calculated by dividing T by S (T/S). Subsequently, the RTL was averaged over the triplicates of each sample, discarding values with a coefficient of variation greater than 0.15.

### 2.3. Statistical Analysis

For the descriptive study, categorical data were analyzed using the chi-squared test, and continuous variables using the Mann–Whitney U test. The generalized linear model (GLM) was used to evaluate the impact of HIV infection (HIV/HCV group vs HCV group) on the telomere length at baseline and at the end of follow-up. This test provides the difference between groups as an arithmetic mean ratio (AMR). GLM tests for independent groups were adjusted for the most relevant covariates, which were selected by a stepwise algorithm (*p* < 0.2). The covariates used were age, sex, body mass index, alcoholism, smoking status, IVDUs, previous HCV treatment, HCV genotype, statin treatment, and decompensation.

Moreover, mixed GLM with gamma distribution (log-link) is used to evaluate repeated measurements. Our model only included two factors: group (HIV/HCV group vs HCV group; or HIV/HCV compensated vs decompensated) and time (baseline vs final). The interaction between group and time was taken into account, generating the statistical models as follows: (a) RTL ~ time (baseline vs final) + group (HIV/HCV-group vs HCV-group) + (time × group) + (1 patient id); (b) RTL ~ time (baseline vs final) + group (decompensated vs compensated-group) + (time × group) + (1| patient id) for HIV/HCV coinfected patients. For both models, the id of the patient was evaluated as random effect. This test gives us the estimation of average RTL in each one of the two factors analyzed.

The optimal sample size for repeated measures in each group was calculated according to the GRANMO sample size calculator (https://www.imim.cat/ofertadeserveis/software-public/granmo/), which established a minimum of 32 samples. Calculates were performed by using the following parameters: standard deviation of differences of 0.02 and a minimum difference to detect of 0.01.

Statistical Package for the Social Sciences (SPSS) 22.0 (SPSS INC, Chicago, IL, USA) was used to perform the statistical analysis. All *p*-values were two-tailed. The statistical significance was defined as *p* ≤ 0.05.

## 3. Results

### 3.1. Patient Characteristics

Ninety patients started the ESCORIAL study (Figure 1), comprised of 60 HIV/HCV-coinfected patients and 30 HCV-monoinfected. Forty-five HIV/HCV coinfected and nine HCV-monoinfected patients completed the follow-up of the study.

Compares to the HCV-monoinfected patients, HIV/HCV-coinfected patients were younger (*p* < 0.001), had a lower body mass index (BMI) (*p* = 0.002), and had been exposed less frequently to interferons (*p* = 0.011). Also, HIV/HCV-coinfected patients were more likely to be men (*p* = 0.011), smokers (*p* = 0.005), prior intravenous drug users (IVDUs) (*p* < 0.001), and alcohol abusers (*p* = 0.005). Additionally, decompensated HIV/HCV-coinfected patients had higher baseline CTP scores (*p* = 0.039) and lower HCV viral loads (*p* = 0.015) (Table 1).

NRTI regimens were used similarly in HIV/HCV-coinfected compensated or decompensated patients (*p* = 0.104).

### 3.2. RTL Comparison between HIV/HCV-Coinfected and HCV-Monoinfected Patients

**Figure 2:**
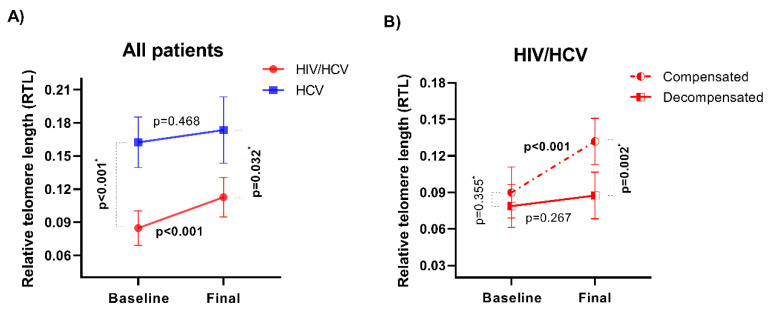
Evolution of the relative telomere length (RTL) in patients with advanced HCV-related cirrhosis after sustained virological response, with all-oral direct-acting antiviral agents (DAAs). (**A**) Comparison of the estimated mean of RTL values in HIV/HCV-coinfected (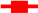) and HCV-monoinfected (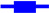) patients (*n* = 60 and *n* = 30 at baseline, *n* = 45 and *n* = 9 throughout follow-up, respectively). (**B**) Comparison of the estimated mean of RTL values in compensated (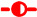) and decompensated (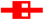) HIV/HCV-coinfected patients (*n* = 35 and *n* = 25 at baseline, *n* = 26 and *n* = 19 throughout follow-up, respectively). The error bars represent the 95% of confidence interval. *p*-values between groups at baseline and 48 weeks after completion of DAA therapy were calculated by an adjusted generalized linear model (GLM) (*), and *p*-values between the two time points by a mixed GLM (see Statistical Analysis section).

The RTL was significantly lower in HIV/HCV-coinfected than in HCV-monoinfected patients, both at baseline (adjusted AMR (aAMR) = 0.60; 95% confidence interval (CI) = 0.46–0.77; *p* < 0.001) and at 48 weeks after completion of HCV therapy (aAMR = 0.69 (95% CI = 0.49–0.97); *p* = 0.032) (Figure 2A; Table 2). Significant variables at baseline were used for adjusting the model, where only sex, previous HCV antiviral treatment, decompensation, and BMI remained as significant co-variates for comparison at baseline, and decompensation and BMI for comparison at 48 weeks. We also explored the relation of RTL with significant variables, such as alcohol intake and smoking status at baseline (*p* = 0.520 and *p* = 0.359, respectively) and at 48 weeks after treatment (*p* = 0.888 and *p* = 0.177, respectively), but no statistically significant differences were found.

### 3.3. Evolution of Telomere Length in HIV/HCV-Coinfected and HCV-Monoinfected Patients

We also assessed the RTL change from baseline up to 48 weeks after completing treatment for both HIV/HCV-coinfected and HCV-monoinfected patients. We found a significant interaction between HIV coinfection and RTL over time, since a significant RTL increase over time was found only for HIV/HCV-coinfected (*p* < 0.001), but not for HCV-monoinfected patients (*p* = 0.468) (Figure 2A, Table 3).

### 3.4. Evolution of Telomere Length in HIV/HCV-Coinfected Patients in Relation to Hepatic Decompensation

We also evaluated RTL values over time between HIV/HCV compensated and decompensated patients (Figure 2B, Table 2). There were no differences in RTL values between compensated and decompensated patients at baseline (aAMR = 0.88 (95% CI = 0.66–1.16); *p* = 0.355), while RTL values were significantly lower in decompensated patients (aAMR = 0.66 (95% CI = 0.51–0.86); *p* = 0.002) at 48 weeks after completing HCV treatment. We also found a significant interaction between decompensation and RTL over time with a mixed GLM, as significant RTL increase over time was found only for compensated (*p* < 0.001), but not for decompensated patients (*p* = 0.267) (Figure 2B; Table 3).

Additionally, we explored if the NRTI regimens with tenofovir (TDF) could have affected the RTL, but no significant differences in the RTLs were found at baseline (aAMR= 1.14 (95% CI = 0.85–1.53); *p* = 0.398) or at 48 weeks (aAMR = 1.02 (95% CI = 0.75–1.38); *p* = 0.892.

## 4. Discussion

The present prospective study shows, for the first time, the evolution of telomere length in the PBMCs of HIV/HCV-coinfected patients after HCV eradication with DAAs. In addition, this is the first study that compares RTL between HIV/HCV-coinfected and HCV-monoinfected patients, showing a clear difference between both groups with advanced HCV-related cirrhosis.

Chronic viral infections promote immune activation, inflammation, and T-cell exhaustion, which accelerate the loss of telomere length in immune cells [2]. In our study, HIV infection had a great impact on telomere length, because HIV/HCV-coinfected patients had lower RTL values in PBMCs than HCV-monoinfected patients, both at baseline and at 48 weeks after DAA therapy, regardless of other clinical and epidemiological factors. HIV triggers a reduction of telomere length [3,4], which may be mitigated by ART [1]. However, this putative protective effect is not enough to compensate for the HIV reduction of telomere length, as RTL values are lower in HIV patients on ART than in healthy controls [16]. According to our results, and the data extracted by Cobos-Jiménez et al. [16], uninfected controls show the higher RTLs, followed by HIV non-viremic, monoinfected patients on ART and HCV patients, and lower RTL data is shown for HIV/HCV-coinfected patients. Regarding antiretroviral therapy, there is a lot of similarity in function between HIV reverse transcriptase and telomerase, which results in telomerase being putatively blocked by NRTIs [5,17,18]. In vivo trials have indicated that TDF is the only NRTI that significantly inhibits telomerase activity and reduces telomere size at therapeutic concentrations [5], although these findings were not confirmed by other studies [19,20]. In this setting, our study is consistent with previous results, according to which TDF treatment had no effect on RTL.

Another remarkable finding was the significant increase in RTL values after HCV eradication with DAAs that was found in HIV/HCV-coinfected patients only. However, a recent study of 24 HCV-monoinfected patients on DAA therapy described a significant telomere elongation in PBMCs 12 weeks after completing HCV treatment [21]. Our HCV-monoinfected patients only showed a slight upward trend in telomere size after HCV therapy that was not significant, probably due to the small sample size at 48 weeks after completing HCV treatment. It may also be relevant that the follow-up time was longer in our study.

The greater increase in RTL values of HIV/HCV-coinfected patients after HCV elimination may be attributed to the fact that this group of patients started with a more immunosuppressed status, and the observed recovery could be greater. Additionally, it is possible that HCV has a greater effect on the shortening of telomeres in HIV/HCV-coinfected patients, and therefore, when HCV is eradicated, a more marked improvement is observed. Zanet et al. [19] found similar results identifying that HCV coinfection in HIV-infected patients can accelerate the shortening of telomeres. Along this same line, Reynoso et al. reported [22] that HIV/HCV coinfection may have a synergistic effect between both HIV and HCV, causing a more pronounced decrease in telomerase activity in HIV/HCV-coinfected patients than in HCV-monoinfected patients. Additionally, note that we previously found no evidences of RTL change in a different cohort of non-cirrhotic patients. However, in this case, patients were treated with IFN [23], whose strong effect might have slowed down the gain in telomere length [24].

Additionally, our data showed that HIV/HCV-coinfected patients with compensated and decompensated cirrhosis showed similar RTL values at baseline. However, after HCV eradication with DAAs, a significant increase in RTL was only observed in compensated patients. When hepatic decompensation occurs in HIV/HCV-coinfected patients, prognosis rapidly worsens and increases the risk of death [25]. Moreover, cirrhosis-associated immune dysfunction is more accentuated in decompensated patients, with higher levels of immune activation, inflammation, and deregulation of the immune system, from which it is more difficult to recover [10]. Under these circumstances, the length of telomeres of immune cells (such as PBMCs) would reach a critical size, which could compromise the telomerase capacity to recover telomere length.

Telomere length change has been previously shown to be different for each cell type of PBMCs [26]. In this context, a similar telomere length has been described for T-cells and monocytes, with longer telomeres for B-cells. Unfortunately, we do not have data on the cellular composition of PBMCs in HCV vs HIV/HCV samples, which would have been interesting in order to check whether different cellular compositions contribute to the RTL differences observed between groups. We only have available data for T-cell subsets in compensated and decompensated groups, which showed no significant differences between groups. However, it has to be noted that the telomere length of PBMCs is correlated with T-cells, B-cells, and monocytes [26], and the change in telomere length with aging is only slightly different for T-cells, B-cells, and monocytes. According to this, PBMCs would reflect the average telomere length of the three populations, being an adequate peripheral marker of telomere size regardless of the cellular composition.

Moreover, the change in the RTL of PBMCs could be extrapolated to the liver, as indicated by Feng et al. [27]. In this work, they observed similar variations in RTL values of paired liver biopsy and PBMC samples from HCV-monoinfected and HBV/HCV-coinfected patients with hepatocellular carcinoma. Therefore, PBMC harvest may be a useful, minimally invasive procedure (liquid biopsy) to estimate RTL in hepatocytes.

Several limitations should be taken into account. Firstly, this is a preliminary study with a limited sample size, which could have limited the possibility of finding statistical significance in some subgroups. However, despite this, note that its longitudinal design allows us a higher statistical power than cross-sectional studies. With regards to this, the sample size to assess RTL change in HIV/HCV-coinfected patients (*n* = 45) is adequate to explore the impact of HCV elimination with DAAs on telomere length in this preliminary study. Secondly, it would be necessary to evaluate telomere length together with other parameters related to senescence, such as cytokine expression, lipid peroxidation, and mitochondrial damage estimation, for a better knowledge of the mechanisms involved in HCV elimination.

## 5. Conclusions

HCV eradication with all-oral DAAs was associated with an increase in telomere length in HIV/HCV-coinfected patients with advanced cirrhosis, particularly in compensated patients. This finding suggests that HCV clearance may have implications in age-related pathologies in this population group.

## Figures and Tables

**Table 1:** Clinical and epidemiological characteristics of patients with advanced HCV-related cirrhosis, stratified by HIV coinfection.

	All Patients			HIV/HCV Patients		
	HIV/HCV	HCV	*p*	Compensated	Decompensated	*p*
No.	60	30		35	25	
Age (years)	51.7 (48.7–53.8)	58.5 (52.3–69.6)	<0.001	51.6 (48.7–53.6)	52.1 (48.7–53.8)	0.887
Gender (male)	49 (81.7%)	17 (56.7%)	0.011	29 (82.9%)	20 (80.0%)	0.778
BMI (kg/m^2^)	23.8 (21.7–26.4)	27.7 (23.1–32.5)	0.002	23.8 (21.2–26.5)	23.5 (21.8–25.0)	0.705
Current smoker	38 (64.4%)	10 (33.3%)	0.005	22 (62.9%)	16 (66.7%)	0.764
Alcohol drinker (>50 g/day)	37 (61.7%)	9 (30%)	0.005	21 (60%)	16 (64.0%)	0.753
Previous IFNα therapy	23 (38.3%)	20 (67%)	0.011	12 (34.3%)	11 (44.0%)	0.445
HCV antiviral therapy						
NS5B	0 (0%)	1 (3.3%)	0.079	0 (0%)	0 (0%)	0.075
NS5A + NS5B	40 (66.7%)	13 (43.3%)		30 (58.8%)	23 (59.0%)	
NS5B + NS3/4A	11(18.3%)	6 (20.0%)		6 (11.8%)	11 (28.2%)	
NS5A + NS5B + NS3/4A	8 (13.3%)	10 (33.3%)		14 (27.5%)	4 (10.3%)	
Unavailable	1 (1.7%)	0 (0%)		1 (2.0%)	0 (0%)	
IVDU	48 (80.0%)	4 (13.3%)	<0.001	27 (77.1%)	21 (84.0%)	0.513
Liver markers						
Child–Pugh–Turcotte	5 (5–5)	5 (5–7)	0.056	5 (5–5)	5 (5–6)	0.039
MELD	9 (8–11)	10 (7–11)	0.608	9 (7–10)	9 (8–12)	0.408
LSM	33.1 (23.6–39.3)	30.7 (27.3–48.0)	0.171	33.3 (26.0–39.3)	31 (18–39.7)	0.382
HVPG	15.3 (12.5–17.3)	16.5 (13–18)	0.467	15.5 (11.5–17.0)	15.3 (13.5–18.0)	0.883
Decompensation	25 (41.7%)	14 (46.7%)	0.652	0 (0%)	25 (100%)	-
HCV markers						
HCV genotype						
1	38 (65.5)	24 (80%)	0.173	21 (60.0%)	17 (73.9%)	0.531
2	0 (0%)	1 (3.3%)		0 (0%)	0 (0%)	
3	9 (15.5%)	3 (10%)		6 (17.1%)	3 (13.0%)	
4	11 (19.0%)	2 (6.7%)		8 (22.9%)	3 (13.0%)	
Log_10_ HCV RNA (IU/mL)	6.2 (5.7–6.7)	6.11 (5.50–6.41)	0.405	6.4 (5.8–6.7)	6.0 (5.3–6.3)	0.015
HIV markers						
Nadir CD4+ T cells	130 (66–245)	-	-	86.5 (40.0–242.0)	150 (99–273)	0.082
Nadir CD4+ T cells < 200 cells/mm^3^	37 (67.3%)	-	-	21 (70.0%)	16 (64.0%)	0.637
Baseline CD4+ T cells	439 (234–717)	-	-	427 (234–721)	444 (227–685)	0.857
Baseline CD4+ T cells < 500 cells/mm^3^	35 (58.3%)	-	-	20 (57.1%)	15 (60.0%)	0.825
Prior AIDS	22 (36.7%)	-	-	12 (34.3%)	10 (40.0%)	0.651
Antiretroviral therapy						
NRTI + NNRTI	7 (11.9%)	-	-	6 (17.1%)	1 (4.2%)	0.104
NRTI + II	31 (52.5%)	-	-	17 (48.6%)	14 (58.3%)	
NRTI + PI	8 (13.6%)	-	-	7 (20.0%)	1 (4.2%)	
PI + II + NNRTI/MVC	4 (6.8%)	-	-	1 (2.9%)	3 (12.5%)	
Others	9 (15.3%)	-	-	4 (11.4%)	5 (20.8%)	

Statistics: Values expressed as absolute number (percentage) and median (interquartile range). *p*-values were calculated by chi-square tests and Mann–Whitney tests. Abbreviations: AIDS, acquired immune deficiency syndrome; BMI, body mass index; HCV, hepatitis C virus; HCV RNA, HCV plasma viral load; HIV, human immunodeficiency virus; HVPG: hepatic venous pressure gradient; LSM, liver stiffness measure; IVDU, intravenous drug user; IFNα, interferon-alpha; MELD, model for end-stage liver disease; NNRTI, non-nucleoside analogue HIV reverse transcriptase inhibitor; NRTI, nucleoside analogue HIV reverse transcriptase inhibitor; PI, protease inhibitor; II, integrase inhibitor, MVC, maraviroc.

**Table 2:** Differences in relative telomeres length of patients, stratified based on HIV coinfection and decompensation.

				Univariable		Multivariable	
		HIV/HCV	HCV	AMR (95% CI)	*p*	aAMR (95% CI)	*p*
All	RTLb	0.08 (0.05–0.14)	0.15 (0.10–0.20)	0.68 (0.55–0.85)	**0.001**	0.60 (0.46–0.77)	**<0.001** **^a^**
	RTL48wk	0.12 (0.07–0.14)	0.17 (0.15–0.18)	0.67 (0.48–0.91)	**0.012**	0.69 (0.49–0.97)	**0.032** **^b^**
		**Decompensated**	**Compensated**	**AMR (95% CI)**	***p***	**aAMR (95% CI)**	***p***
HIV/HCV	RTLb	0.08 (0.06–0.10)	0.08 (0.05–0.18)	0.88 (0.66–1.17)	0.382	0.88 (0.66–1.16)	0.355
	RTL48wk	0.07 (0.04–0.12)	0.13 (0.09–0.16)	0.66 (0.51–0.86)	**0.002**	0.66 (0.51–0.86)	**0.002**

Statics: *p*-values were calculated using univariate and multivariate regression models, adjusted by the clinical and epidemiological characteristics (see Statistical Analysis section), selected by stepwise algorithm. The co-variates that remained in the model were ^a^ sex, previous HCV treatment, liver decompensation, and BMI for comparison at baseline (RTLb); and ^b^ liver decompensation and BMI for comparison at 48 weeks (RTL48wk). The statistically significant differences are shown in bold. Abbreviations: RTL, relative size of telomeres; b, baseline; 48 wk, 48 weeks; *p*-value, level of significance; AMR, arithmetic mean ratio; aAMR, adjusted arithmetic mean ratio; 95% CI, 95% of confidence interval; HCV, hepatitis C virus; HIV, human immunodeficiency virus.

**Table 3:** Mean differences in RTL at baseline and 48 weeks after completing HCV treatment. Patients were stratified based on HIV coinfection and decompensation (mixed GLMs).

	Baseline	48wk	DM (ES)	*p*
All	0.12 (0.01)	0.14 (0.01)	−0.02(0.01)	**0.001**
HIV/HCV	0.08 (0.01)	0.11 (0.01)	−0.03 (0.01)	**<0.001**
HCV	0.16 (0.01)	0.17 (0.11)	−0.01 (0.01)	0.468
HIV/HCV patients	0.08 (0.01)	0.11 (0.01)	−0.02 (0.01)	**<0.001**
Compensated	0.09 (0.01)	0.13 (0.01)	−0.04 (0.01)	**<0.001**
Decompensated	0.08 (0.01)	0.09 (0.01)	−0.01 (0.01)	0.267

Statistics: The values for baseline and 48 weeks are shown as mean and standard error. *p* values were calculated using mixed generalized linear models (GLMs). Statistically significant differences are shown in bold. Abbreviations: RTL. relative telomere size; HCV, hepatitis C virus; HIV, human immunodeficiency virus; *p*, level of significance; DM, difference of means; ES, standard error.

## Abbreviations

CHC	Chronic hepatitis C
cART	Combination antiretroviral therapy
DAAs	Direct-acting antivirals
GLM	Generalized linear model
HCV	Hepatitis C virus
HCC	Hepatocellular carcinoma
HIV	Human immunodeficiency virus
IFN	Interferon
LSM	Liver stiffness measurement
RTL	Relative telomere length

## Appendix A

The ESCORIAL study group:

Hospital General Universitario Gregorio Marañón (Madrid, Spain): Cristina Díez, Luis Ibáñez, Leire Pérez-Latorre, Diego Rincón, Teresa Aldámiz-Echevarría, Vega Catalina, Pilar Miralles, Teresa Aldámiz-Echevarría, Francisco Tejerina, María C Gómez-Rico, Esther Alonso, José M Bellón, Rafael Bañares, and Juan Berenguer.

Hospital Universitario La Paz/IdiPAZ (Madrid, Spain): José Arribas, José I Bernardino, Carmen Busca, Javier García-Samaniego, Víctor Hontañón, Luz Martín-Carbonero, Rafael Micán, María L Montes-Ramírez, Victoria Moreno, Antonio Olveira, Ignacio Pérez-Valero, Eulalia valencia, and Juan González-García.

Hospital Universitario Puerta de Hierro (Madrid, Spain): Elba Llop and José Luis Calleja.

Hospital Universitario Ramón y Cajal (Madrid, Spain): Javier Martínez and Agustín Albillos.

Fundación SEIMC/GeSIDA (Madrid, Spain): Marta de Miguel, María Yllescas, and Herminia Esteban.
